# Comparing Surgical Outcomes of Laparoscopic Myomectomy Versus Laparotomy at a Specialized Tertiary Hospital for Women’s Health: A Retrospective Study

**DOI:** 10.7759/cureus.117980

**Published:** 2026-10-15

**Authors:** Hala A Bokhari, Maysoon Adham, Maha Alruwaythi, Sara Almutairi, Sarah Alghuraybi, Sara Ibrahim, Wafa Faried

**Affiliations:** 1 College of Medicine, Ministry of National Guard Health Affairs, Riyadh, SAU; 2 Obstetrics and Gynecology, Ministry of National Guard Health Affairs, Riyadh, SAU; 3 Obstetrics and Gynecology, King Abdulaziz Medical City, Riyadh, SAU; 4 Obstetrics and Gynecology, King Saud bin Abdulaziz University for Health Sciences, Riyadh, SAU; 5 College of Medicine, Princess Nourah University, Riyadh, SAU

**Keywords:** hospital length of stay, laparoscopic myomectomy, laparotomy, open myomectomy, operative time, post-operative complications, retrospective cohort study, surgical outcomes, uterine fibroids

## Abstract

Background: Uterine fibroids, which are benign tumors, are the most frequent tumors in women of reproductive age. Although myomectomy, the surgical removal of fibroids, is the most suitable option from the perspective of preserving the patient's reproductive potential, there is not enough real-world research known in Saudi Arabia on which of the two surgical techniques, laparoscopic or open (abdominal) myomectomy.

Aim: To assess and compare surgical outcomes, such as intraoperative blood loss, duration of hospital stays, postoperative complications, and duration of surgery, of laparoscopic myomectomy to open abdominal myomectomy (laparotomy) for women suffering from fibroids at the only specialized tertiary women's hospital in Saudi Arabia.

Methods: This real-world retrospective study analyzes 299 women who underwent myomectomy from January 2021 to October 2025. Participants were grouped according to laparoscopic (n = 88) and open (n = 211) myomectomy. Women diagnosed with uterine fibroids, women aged 18 to 45, and women who underwent either laparoscopic or open myomectomy were included in the study. This study excluded patients with missing or incomplete data, patients with suspected malignant uterine tumors, and postmenopausal women. Comparisons of demographics and surgical outcomes were performed with the Mann-Whitney U test, independent t-test, and chi-square and Fisher’s exact tests, as appropriate. Effect sizes were calculated using the rank-biserial correlation coefficient with bootstrap 95% confidence intervals (10,000 iterations). Age, body mass index, fibroid size, and fibroid multiplicity were adjusted for in the evaluation of the independent association between surgical approach and outcomes. Body mass index and fibroid count were the criteria for pre-specified subgroup analysis.

Results: The two groups revealed no statistical differences in age (median 37 vs. 37 years; p = 0.901) and body mass index (median 27.6 vs. 28.5 kg/m²; p = 0.625). Patients undergoing laparoscopic myomectomy had significantly shorter length of hospital stay (median 3.0 vs. 4.0 days; p < 0.001; rank-biserial r = 0.45); this difference remained after multivariable analysis (β = −0.860; 95% CI: −1.251 to −0.469; p < 0.001). On the contrary, laparoscopic myomectomy had a significantly greater average surgery duration (median 213.0 vs. 121.0 minutes; p < 0.001; rank-biserial r = −0.527; adjusted β = 90.490; 95% CI: 70.029-110.951; p < 0.001). There were no significant differences in estimated blood loss (median 300 vs. 400 mL; p = 0.394), hemoglobin drop (median 13.0 vs. 14.0 g/L; p = 0.948), and overall complications (3.4% vs. 1.4%; p = 0.364). On multivariable logistic regression analysis, laparoscopic myomectomy was not a significant complication predictor (OR = 1.715; 95% CI: 0.283-10.378; p = 0.557). Larger and more numerous fibroids were associated with longer surgery and greater blood loss.

Conclusion: Compared to open myomectomy, laparoscopic myomectomy has meaningfully shorter hospital stays, with similar rates of blood loss, decline in hemoglobin, and similar rates of postoperative complications. The downside is that laparoscopic myomectomy has a substantially longer operative time. Laparoscopic myomectomy is safe and effective in appropriately selected patients at specialized tertiary care centers. Fibroid size and number, and not the choice of surgical technique, were the main contributors to intraoperative blood loss.

## Introduction

Uterine fibroids, also medically known as leiomyomas, are defined as benign monoclonal tumors that arise from the myometrium of the uterus [1]. Internationally, the incidence of uterine fibroids is highest in women of the ages between 35-39 [2]. Fibroids vary in presentation, as some patients are asymptomatic while others report symptoms such as heavy bleeding, pelvic pressure, increased urinary frequency, constipation, and lower back pain [3]. The treatment for uterine fibroids depends on the clinical presentation of the patient, with monitoring recommended for those who are asymptomatic [3]. This is primarily due to the possibility of tumor shrinkage during menopause [3]. For symptomatic patients, medications including hormonal contraceptives, non-steroidal anti-inflammatory drugs, and tranexamic acid are used to treat menorrhagia [3]. Definitive treatment includes surgical operations, namely myomectomy, hysterectomy, and uterine artery embolization [3]. Although hysterectomy is indicated for fibroids, myomectomy is the preferred treatment for patients who have the desire to conceive [4].

Myomectomy can be performed either by laparoscopic surgery or by laparotomy, with some studies done to compare the outcomes of the two surgical approaches [5]. One study compared two groups of patients who underwent both procedures and found that those who received the laparoscopic myomectomy had significantly less blood loss than those who received the laparotomy [5]. Another study showed that laparoscopic myomectomy resulted in a shorter hospital stay [6]. Even though laparoscopic surgery resulted in better outcomes for the patients, an increase in the duration of the surgery has been reported [7].

Within a single-center retrospective study, the study found that laparoscopic myomectomies were considered a better alternative to laparotomy. However, laparoscopic myomectomies require longer surgical duration and surgical teams with high expertise. The study also found that the success rate of laparoscopic myomectomy correlates with the number of uterine fibroids, which highlights the impact of the number of uterine fibroids [8].

In a study done in Saudi Arabia that investigated the complications associated with a myomectomy, it showed that the most common incidents were hemorrhage, followed by pulmonary embolism and urinary tract infections. The study also found laparoscopy to be a more effective choice when it comes to surgery duration, hospital stay, and prevalence of complications [9].

Although there are extensive studies done on myomectomies, they still require further research in Saudi Arabia, with an emphasis on the outcomes experienced by Saudi women following both types of surgeries. This study is designed to compare the surgical outcomes between laparoscopic myomectomy and laparotomy in women with uterine fibroids by assessing hemoglobin pre- and post-operation, the amount of blood loss, operation duration, and the length of stay post-operation. In addition, the occurrence of postoperative complications, such as surgical site infections, small bowel obstruction, and fever. 

## Materials and methods

This retrospective cohort study was conducted at the Specialized Hospital for Women’s Health at National Guard Health Affairs, located in Riyadh, Saudi Arabia. King Abdullah International Medical Research Center issued approval 00000217325. Women with the diagnosis of uterine fibroids, women of the ages 18 to 45, and women who underwent either laparoscopic or open myomectomy were included in the study. This study excluded patients with missing or incomplete data, patients with suspected malignant uterine tumors, and postmenopausal women.

Statistical analyses were conducted in SAS 9.4 (SAS Institute Inc., Cary, NC, US) after data cleaning in Microsoft Excel (Microsoft Corporation, Redmond, Washington, United States). The Shapiro-Wilk test was used to determine the distribution of continuous variables, with a significance level of α = 0.05. Variables that deviated from normal distributions were summarized as medians with interquartile ranges (IQR) and were compared between the laparoscopic and open myomectomy groups using the Mann-Whitney U test. Normally distributed variables were summarized as means ± standard deviations (SD), and comparisons were made using the independent-samples Student's t-test. For categorical variables, frequencies and percentages were summarized, and comparisons were made using the Pearson chi-square (χ²) test. If the expected cell count in any cell of the contingency table was less than five, Fisher's exact test was used in place of the chi-square test.

Changes in hemoglobin levels from the preoperative to postoperative periods were analyzed using the Wilcoxon signed-rank test because the hemoglobin difference values were not normally distributed. For each patient, the hemoglobin drop was defined as the arithmetic difference between the preoperative and postoperative hemoglobin values. However, the rank-biserial correlation coefficient (r) from the Mann-Whitney U statistic was used to quantify effect sizes for between-group comparisons of continuous outcomes. Effect sizes were described as negligible (|r| < 0.1), small (0.1-0.3), moderate (0.3-0.5), and large (>0.5). Bootstrap resampling with 10,000 iterations was used to obtain bias-corrected and accelerated 95% confidence intervals (95% CI) for the median differences between the groups.

The association between the type of surgery (laparoscopic and open surgery, with open surgery as the reference category) and continuous outcomes of surgery, that is, length of stay in hospital, duration of surgery, volume of blood lost during surgery, and drop in hemoglobin, was examined using multivariable linear regression as a method. The models were adjusted for clinically important a priori confounding variables, including age (years), body mass index (kg/m²), largest fibroid size (cm), and number of fibroids (single vs. multiple). The results of the study were expressed as unstandardized β coefficients and 95% confidence intervals. The coefficient of determination (R²) and the overall F-test were used to measure model fit.

Multivariable logistic regression was conducted to assess the independent association between surgery type and surgery complications. Although the same covariates were included in the linear regression models, the results are presented as adjusted odds ratios (ORs) with 95% confidence intervals (CIs). None of the covariates were statistically significant since the event rate is low.

Due to the non-normal distribution of most of the mentioned variables, Spearman's rank correlation coefficients (ρ) were used to examine bivariate associations in some of the most clinically relevant continuous variables, which included the duration of the operation, estimated blood loss, the size of the fibroid, length of stay in the hospital, and the drop in the level of hemoglobin. Moreover, to aid understanding of the operationalized key findings across clinically important strata, we conducted a pre-specified subgroup analysis. The primary outcomes of interest, including length of hospital stay, duration of the procedure, estimated blood loss, and change in hemoglobin levels, were compared between the laparoscopic and open groups in the following strata: body mass index (BMI <25 kg/m² normal/underweight vs. BMI ≥25 kg/m² overweight/obese) and fibroid count (single vs. multiple (≥2)). All subgroup analyses were conducted using the Mann-Whitney U test. A qualitative assessment of the consistency of the findings was conducted by evaluating the direction and statistical significance of the subgroup-specific estimates relative to the overall estimates.

For the years 2021 to 2025, temporal trends in the use of laparoscopic versus open myomectomy were assessed by calculating the annual percentage of laparoscopic myomectomies. Annual volumes for each procedure, as well as the average rate of laparoscopic myomectomy utilization for the entire study period, were calculated. Moreover, sample size calculations were not performed for this retrospective study. A two-sided p-value less than 0.05 was considered statistically significant for all analyses.

## Results

Study population and baseline characteristics

We identified 299 women who had a myomectomy between January 2021 and October 2025. There were 88 (29.4%) women who had a laparoscopic myomectomy and 211 (70.6%) who had an open abdominal myomectomy (laparotomy). The baseline characteristics (demographic and clinical) of the study participants are shown in Table 1. However, both groups had similar ages, with the laparoscopic group having a median age of 37 years (IQR: 32.8-41.00) and the open group having a median age of 37 years (IQR: 33.00-40.00) (p = 0.901). The groups were also similar in body mass index (median 27.62 vs. 28.48 kg/m²; p = 0.625). Height was the only baseline variable that was normally distributed, with mean heights of 157.05 ± 5.40 cm and 156.88 ± 6.19 cm in the laparoscopic and open groups, respectively (p = 0.814). The distribution of BMI categories did not differ significantly between groups (χ² test, p = 0.638; Figure 1), confirming adequate group matching for body habitus. Moreover, statistically significant differences were observed in specific fibroid characteristics. The largest fibroid measurement was greater in the laparoscopic group (median 8.0 cm, IQR: 6.15-12.00 vs. 7.0 cm, IQR: 5.40-9.00; p = 0.013), while the fibroids’ count also differed between groups (χ² = 7.16, p = 0.028), with the open myomectomy group having larger percentages of patients with three or more fibroids 88 (41.7%) vs. 22 (25.3%). This may indicate some patterns of clinical decision-making that favor open surgery in more complex cases.

**Table 1:** Baseline demographics and clinical characteristics of study participants BMI: body mass index; IQR: interquartile range; SD: standard deviation; D1: dimension 1; D2: dimension 2 Continuous variables are reported as median (IQR) with the Mann-Whitney U test, or mean ± SD with the independent t-test, based on Shapiro-Wilk normality assessment (α = 0.05). Categorical variables are compared using the chi-square test. * p < 0.05

Variable	Total (N = 299)	Laparoscopic (n = 88)	Open (n = 211)	Test Statistic	P-value
Continuous variables, median (IQR) or mean ± SD					
Age (years), median (IQR)	37.0 (33.0–41.0)	37.0 (32.8–41.0)	37.0 (33.0–40.0)	U = 9,213	0.901
Height (cm), mean ± SD	156.9 ± 6.0	157.1 ± 5.4	156.9 ± 6.2	t = 0.24	0.814
Weight (kg), median (IQR)	68.5 (59.0–80.0)	68.2 (59.0–80.0)	68.6 (59.4–80.0)	U = 9,261	0.954
BMI (kg/m²), median (IQR)	28.1 (24.2–33.1)	27.6 (24.1–32.8)	28.5 (24.4–33.3)	U = 8,978	0.625
Fibroid size D1 (cm), median (IQR)	7.5 (5.9–10.0)	8.0 (6.2–12.0)	7.0 (5.4–9.0)	U = 10,958	0.013*
Fibroid size D2 (cm), median (IQR)	6.0 (4.0–8.0)	5.0 (3.4–7.6)	6.0 (4.7–8.0)	U = 7,266	0.005*
Categorical variables, n (%)					
BMI Category	-	-	-	χ² = 3.41	0.638
Underweight	7 (2.3)	2 (2.3)	5 (2.4)	-	-
Normal weight	82 (27.4)	25 (28.4)	57 (27.0)	-	-
Overweight	97 (32.4)	27 (30.7)	70 (33.2)	-	-
Obesity Class I	60 (20.1)	22 (25.0)	38 (18.0)	-	-
Obesity Class II	40 (13.4)	8 (9.1)	32 (15.2)	-	-
Obesity Class III	13 (4.3)	4 (4.5)	9 (4.3)	-	-
Number of Fibroids	-	-	-	χ² = 7.16	0.028*
Single	155 (51.8)	54 (62.1)	101 (47.9)	-	-
Two	33 (11.0)	11 (12.6)	22 (10.4)	-	-
Three or more	110 (36.8)	22 (25.3)	88 (41.7)	-	-

**Figure 1:**
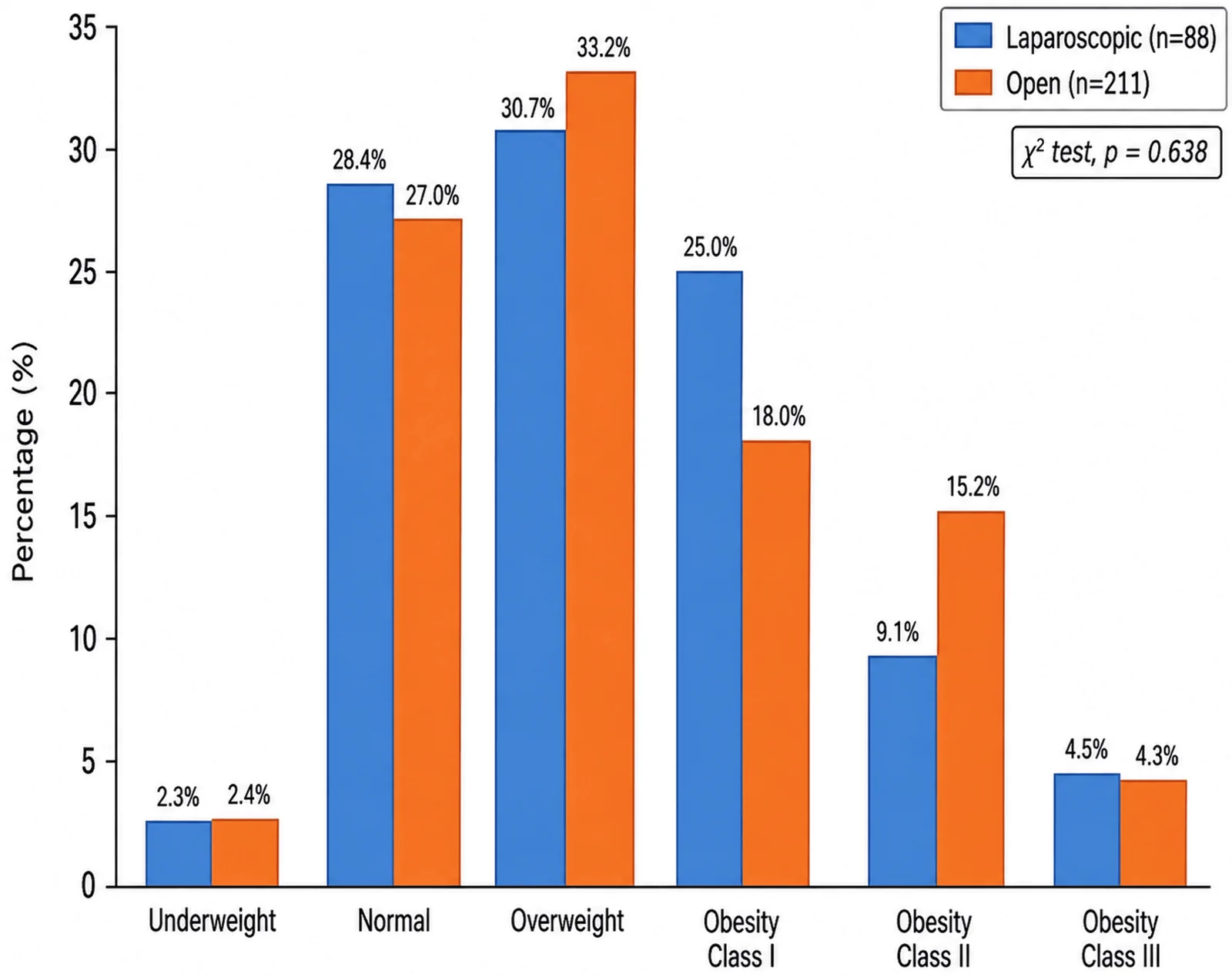
Distribution of BMI categories by myomectomy type

Blood loss and hemoglobin changes (Objective 1)

Preoperative hemoglobin values were similar in the laparoscopic and open groups (median 123.0 g/L, IQR: 108.5-130.5 vs. 117.0 g/L, IQR: 105.0-128.0; p = 0.098), and postoperative values were also similar (median 107.0 vs. 103.0 g/L; p = 0.149). Of note, the hemoglobin drop (calculated as preoperative hemoglobin minus postoperative hemoglobin) was almost the same in both groups (median 13.0 g/L, IQR: 1.25-22.00 vs 14.0 g/L, IQR: 5.00-22.00, p = 0.948), suggesting that the surgical approach does not have a unique effect on hemoglobin reduction (Tables 2, 3; Figure 2D).

**Table 2:** Comparison of surgical outcomes between laparoscopic and open myomectomy All values are reported as median (IQR). Between-group comparison by Mann-Whitney U test. *** p < 0.001. Hgb: hemoglobin; IQR: interquartile range; Op: operation

Outcome Variable	Total (N = 299)	Laparoscopic (n = 88)	Open (n = 211)	U Statistic	P-value
Pre-op Hgb (g/L)	119.0 (105.0–129.0)	123.0 (108.5–130.5)	117.0 (105.0–128.0)	10,411	0.098
Post-op Hgb (g/L)	104.0 (93.0–114.0)	107.0 (96.0–115.8)	103.0 (92.0–113.0)	10,042	0.149
Hemoglobin drop (g/L)	14.0 (4.0–22.0)	13.0 (1.3–22.0)	14.0 (5.0–22.0)	9,029	0.948
Blood loss (mL)	400.0 (200.0–500.0)	300.0 (200.0–500.0)	400.0 (200.0–500.0)	7,461	0.394
Length of stay (days)	4.0 (3.0–5.0)	3.0 (2.0–4.0)	4.0 (3.0–5.0)	5,109	<0.001***
Op duration (min)	140.0 (105.0–205.5)	213.0 (143.8–300.0)	121.0 (96.5–165.5)	14,177	<0.001***

**Table 3:** Within-group pre- vs. post-operative hemoglobin paired analysis Wilcoxon signed-rank test for within-group paired comparison. Δ = Pre-op Hgb minus Post-op Hgb. *** p < 0.001.

Group	Pre-op Hgb Median (IQR)	Post-op Hgb Median (IQR)	Median Δ (IQR)	Test	P-value
Laparoscopic	123 (109–131)	107 (96–116)	13 (1–22)	Wilcoxon	<0.001***
Open	117 (105–128)	103 (92–113)	14 (5–22)	Wilcoxon	<0.001***

**Figure 2:**
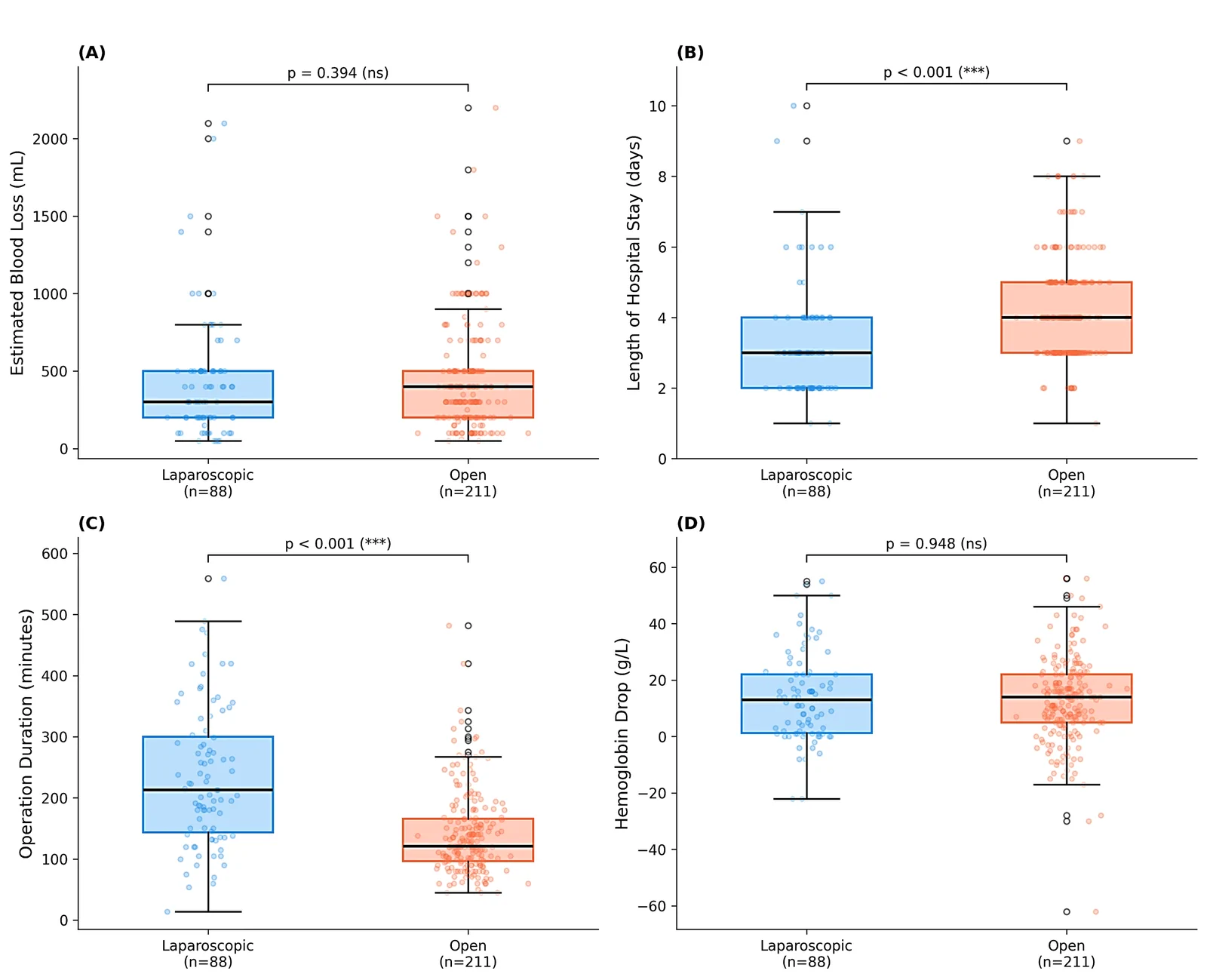
Comparison of surgical outcomes between laparoscopic and open myomectomy groups A: estimated blood loss (mL); B: length of hospital stay (days); C: operation duration (minutes); D: hemoglobin drop (g/L). Box plots show the median, IQR, and individual data points. P-values are from the Mann-Whitney U test. ns: not significant; *** p < 0.001

Using the within-group unpaired McNemar's test, both laparoscopic (median Δ = 13 g/L; p < 0.001) and open (median Δ = 14 g/L; p < 0.001) groups showed a statistically significant drop in postoperative hemoglobin levels, as illustrated in Figure 3. The magnitude of decline, however, was comparable across both groups.

**Figure 3:**
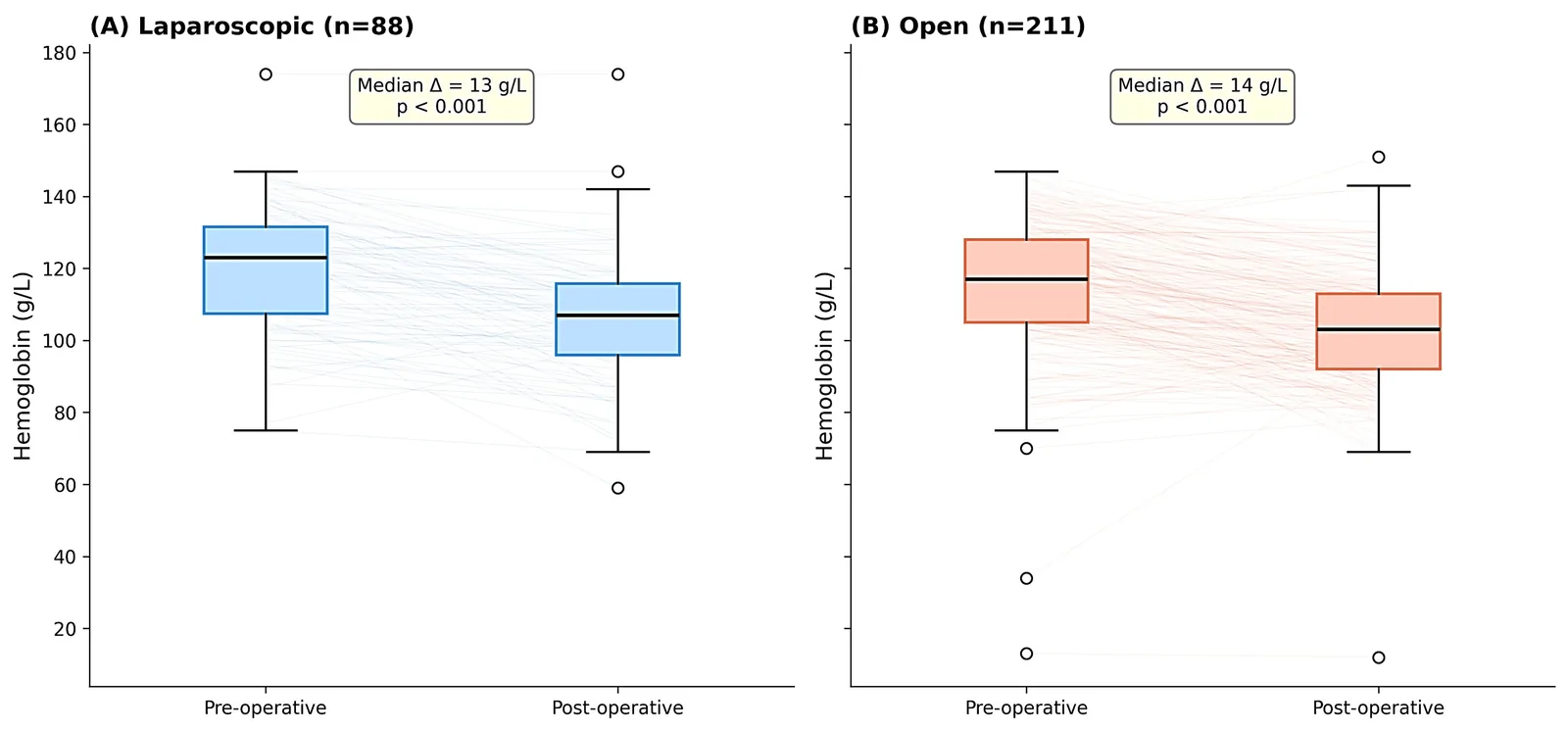
Preoperative vs. postoperative hemoglobin levels by myomectomy type A: laparoscopic group (n = 88); B: open group (n = 211). Lines connect individual patient-pair values. P-values from the Wilcoxon signed-rank test; p < 0.001. Δ: median difference.

For estimated iatrogenic blood loss, laparoscopic (median = 300 mL, IQR: 200-500) and open (median = 400 mL, IQR: 200-500) groups showed no significant difference (p = 0.394; Figure 2A).

Hospital length of stay (Objective 2)

Patients who had laparoscopic myomectomies had a statistically significantly shorter hospital stay than patients who had open myomectomies (median 3.0 days, IQR: 2.0-4.0 vs. 4.0 days, IQR: 3.0-5.0; p < 0.001; Table 2; Figure 2B), with a large effect size (rank-biserial r = 0.45), and a bootstrap 95% confidence interval for the median difference of −1.0 days (95% CI: −1.0 to −1.0; Table 4). After controlling for age, BMI, size, and number of fibroids, the laparoscopic approach was associated with 0.86 fewer hospital days (β = −0.860, 95% CI: −1.251 to −0.469; p < 0.001; Table 5; Figure 4).

**Table 4:** Effect sizes and 95% bootstrap confidence intervals for between-group comparisons Rank-biserial r: effect size from Mann-Whitney U. |r| < 0.1 = negligible; 0.1–0.3 = small; 0.3–0.5 = moderate; > 0.5 = large. 95% CI for median difference computed via 10,000-iteration bootstrap resampling. Negative difference favors laparoscopic. *** p < 0.001

Outcome	Lap Median	Open Median	Median Diff	95% CI Lower	95% CI Upper	Rank- Biserial r	P-value
LOS (days)	3.0	4.0	−1.0	−1.0	−1.0	0.450	<0.001***
Op duration (min)	213	121	+92.0	62.0	127.0	−0.527	<0.001***
Blood loss (mL)	300	400	−100	−100	100	0.065	0.394
Hgb drop (g/L)	13.0	14.0	−1.0	−6.0	4.0	0.005	0.948
Pre-op Hgb (g/L)	123	117	+6.0	−0.5	10.5	−0.121	0.098
Post-op Hgb (g/L)	107	103	+4.0	−2.0	9.0	−0.107	0.149

**Table 5:** Multivariable linear regression analysis of surgical outcomes Reference: Open myomectomy, single fibroid. β = adjusted change in outcome per unit increase in the predictor (or for the category vs. the reference). All models were simultaneously adjusted for age, BMI, fibroid size (largest dimension), and fibroid multiplicity. * p < 0.05; ** p < 0.01; *** p < 0.001

Outcome / Predictor	β Coefficient	95% CI Lower	95% CI Upper	P-value	R²	Model F p-value
Length of Hospital Stay (days)	-	-	-	-	0.095	<0.001
Laparoscopic (vs. Open)	−0.860	−1.251	−0.469	<0.001***	-	-
Age (years)	0.002	−0.030	0.034	0.922	-	-
BMI (kg/m²)	0.005	−0.022	0.032	0.719	-	-
Fibroid size (cm)	−0.003	−0.044	0.037	0.872	-	-
Multiple fibroids	0.386	0.040	0.733	0.029*	-	-
Operation Duration (min)	-	-	-	-	0.374	<0.001
Laparoscopic (vs. Open)	90.490	70.029	110.951	<0.001***	-	-
Age (years)	1.223	−0.453	2.900	0.152	-	-
BMI (kg/m²)	0.750	−0.686	2.186	0.305	-	-
Fibroid size (cm)	6.952	4.825	9.079	<0.001***	-	-
Multiple fibroids	45.256	27.104	63.408	<0.001***	-	-
Estimated Blood Loss (mL)	-	-	-	-	0.110	<0.001
Laparoscopic (vs. Open)	−34.044	−126.771	58.683	0.470	-	-
Age (years)	0.693	−6.906	8.292	0.858	-	-
BMI (kg/m²)	8.267	1.758	14.776	0.013*	-	-
Fibroid size (cm)	21.686	12.047	31.325	<0.001***	-	-
Multiple fibroids	125.340	43.076	207.604	0.003**	-	-
Hemoglobin Drop (g/L)	-	-	-	-	0.036	0.073
Laparoscopic (vs. Open)	−0.480	−4.403	3.443	0.810	-	-
Age (years)	−0.025	−0.347	0.296	0.876	-	-
BMI (kg/m²)	−0.239	−0.514	0.037	0.089	-	-
Fibroid size (cm)	0.512	0.104	0.920	0.014*	-	-
Multiple fibroids	2.218	−1.263	5.699	0.211	-	-

**Figure 4:**
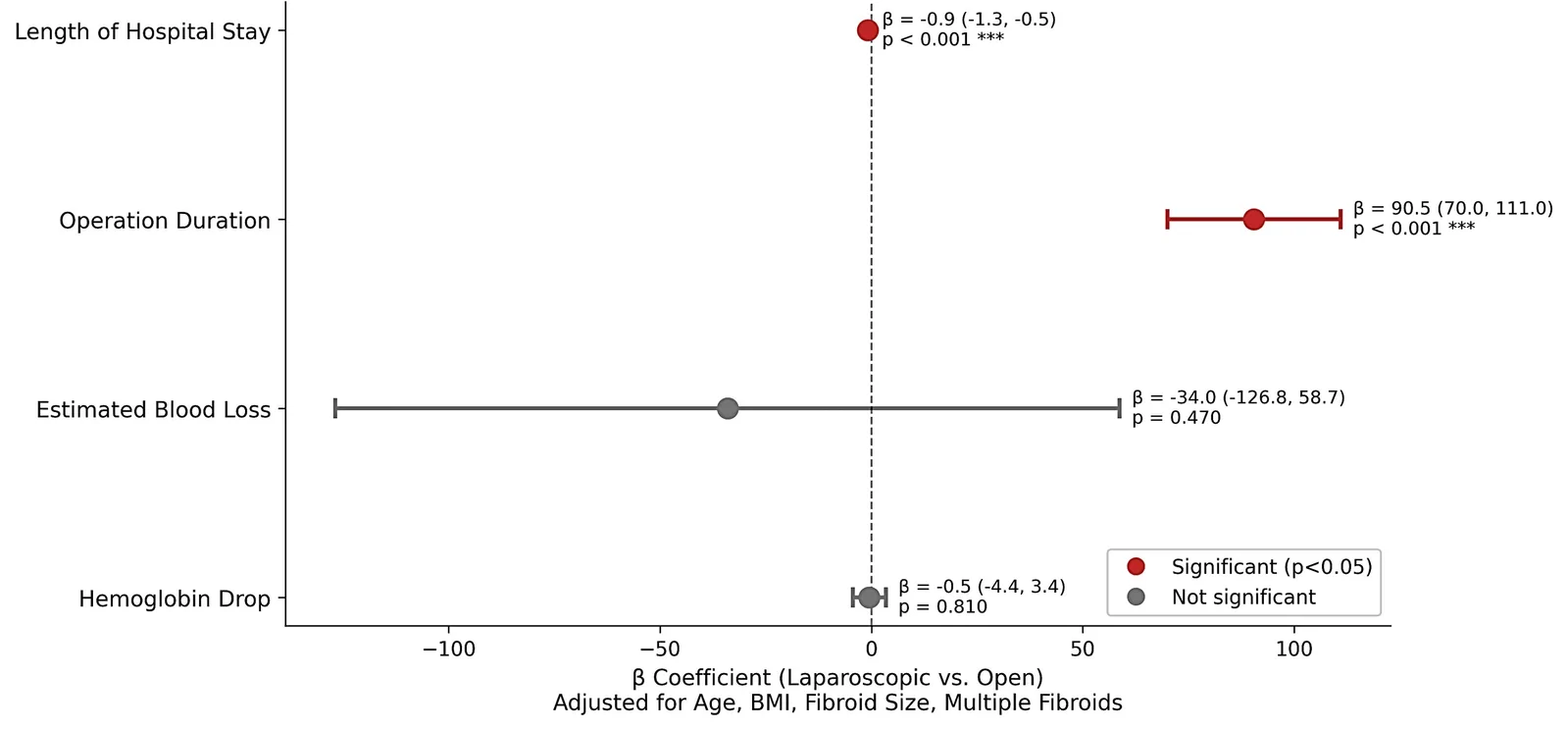
Forest plot of adjusted β coefficients for the laparoscopic approach across four surgical outcomes (multivariable linear regression) Models adjusted for age, BMI, fibroid size, and fibroid multiplicity. Error bars represent 95% confidence intervals. Red markers indicate statistical significance (p < 0.05).

This result was reiterated in the subgroup analyses. The laparoscopic advantage for length of stay was consistent among all subgroups, including those with normal/underweight BMI (3.0 vs. 4.0 days; p < 0.001), those who were overweight/obese (3.0 vs. 4.0 days; p < 0.001), those with single fibroids (3.0 vs. 4.0 days; p < 0.001), and those with multiple fibroids (3.0 vs. 4.0 days; p < 0.001; Table 6).

**Table 6:** Subgroup analyses of key outcomes by BMI category and fibroid number The Mann-Whitney U test was used for all subgroup comparisons. Consistent = significant in the subgroup in the same direction as the overall finding; *** p < 0.001

Subgroup	Outcome	Laparoscopic Median (n)	Open Median (n)	Median Difference	P-value	Consistency
Normal/Underweight (BMI < 25)						
-	LOS (days)	3.0 (27)	4.0 (62)	−1.0	<0.001***	Consistent
-	Op duration (min)	224.0 (27)	128.5 (62)	+95.5	<0.001***	Consistent
-	Blood loss (mL)	300.0 (24)	400.0 (57)	−100	0.073	Attenuated
Overweight/Obese (BMI ≥ 25)						
-	LOS (days)	3.0 (61)	4.0 (149)	−1.0	<0.001***	Consistent
-	Op duration (min)	213.0 (61)	121.0 (149)	+92.0	<0.001***	Consistent
-	Blood loss (mL)	400.0 (57)	350.0 (132)	+50	0.892	Attenuated
Single Fibroid						
-	LOS (days)	3.0 (54)	4.0 (101)	−1.0	<0.001***	Consistent
-	Op duration (min)	202.5 (54)	109.0 (101)	+93.5	<0.001***	Consistent
-	Blood loss (mL)	300.0 (50)	300.0 (92)	0	0.380	Attenuated
-	Hgb drop (g/L)	11.5 (53)	11.0 (100)	+0.5	0.591	Attenuated
Multiple Fibroids						
-	LOS (days)	3.0 (33)	4.0 (110)	−1.0	<0.001***	Consistent
-	Op duration (min)	244.0 (33)	141.5 (110)	+102.5	<0.001***	Consistent
-	Blood loss (mL)	400.0 (32)	400.0 (96)	0	0.134	Attenuated
-	Hgb drop (g/L)	14.0 (32)	15.5 (109)	−1.5	0.471	Attenuated

Postoperative complications (Objective 3)

In both groups, the overall incidence of complications was low (Table 7, Figure 5). In the laparoscopic group, 1 (1.1%) patient developed postoperative fever, whereas in the open group, 3 (1.4%) patients did. This was considered statistically insignificant (Fisher's exact test, p = 1.000). Surgical site infection occurred in two (2.3%) patients in the laparoscopic group and in none in the open group; although the rate was higher in the laparoscopic group, the difference was not statistically significant (Fisher's exact test, p = 0.086). No early postoperative small bowel obstructions were reported in either group. Overall, the complication rate (any complication) was 3.4% in the laparoscopic group and 1.4% in the open group (p = 0.364).

**Table 7:** Comparison of postoperative complications between laparoscopic and open myomectomy Fisher’s exact test was used due to expected cell counts <5. † No events were observed in either group; statistical testing was not applicable. Any complication = presence of at least one of: postoperative fever, surgical site infection, or small bowel obstruction.

Complication	Total (N = 299) n (%)	Laparoscopic (n = 88) n (%)	Open (n = 211) n (%)	Test	P-value
Postoperative fever	4 (1.3)	1 (1.1)	3 (1.4)	Fisher’s exact	1.000
Surgical site infection	2 (0.7)	2 (2.3)	0 (0.0)	Fisher’s exact	0.086
Small bowel obstruction	0 (0.0)	0 (0.0)	0 (0.0)	N/A†	N/A
Any complication	6 (2.0)	3 (3.4)	3 (1.4)	Fisher’s exact	0.364

**Figure 5:**
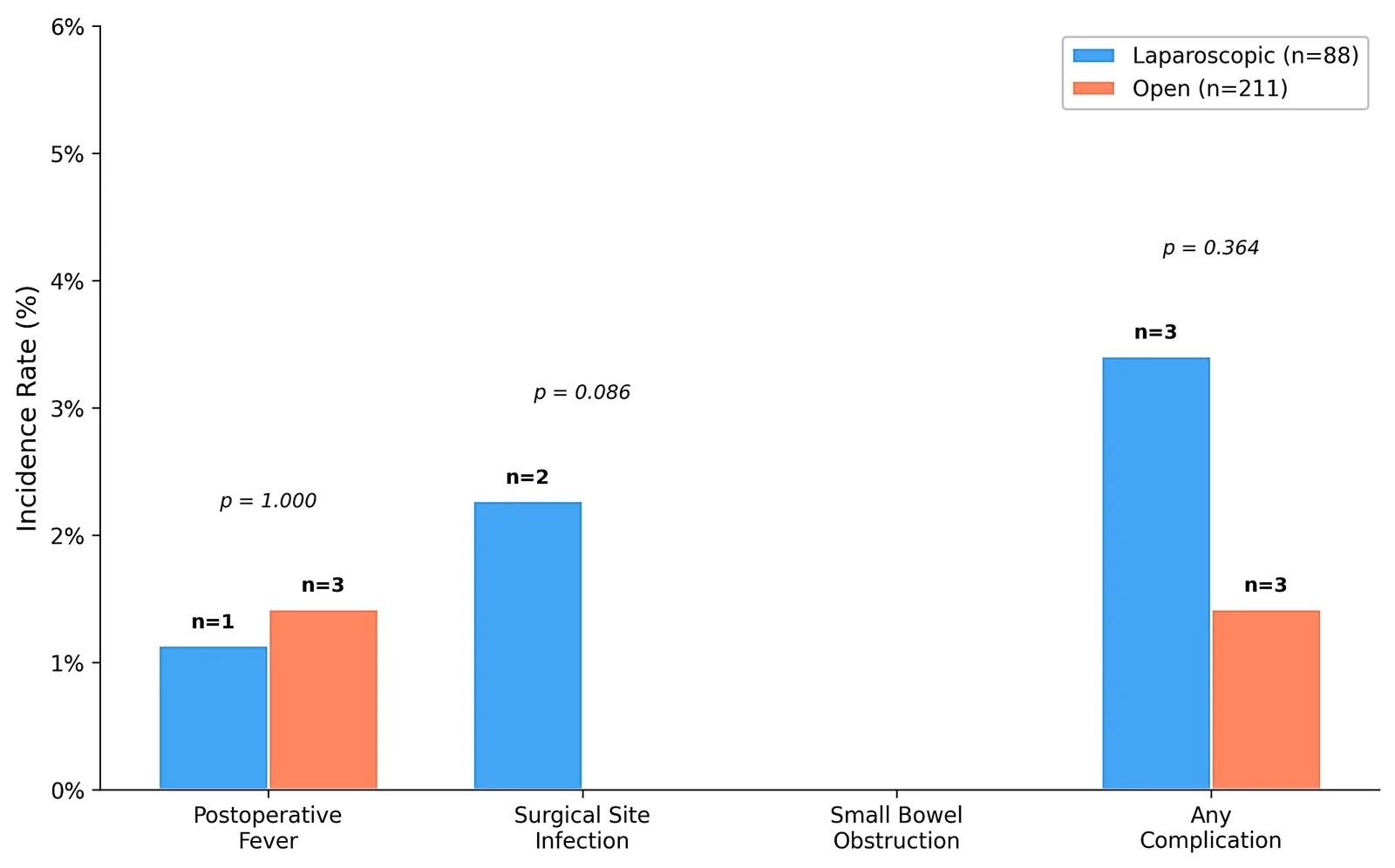
Incidence of postoperative complications by myomectomy type No cases of small bowel obstruction were observed in either group. P-value from Fisher’s exact test; p = 0.364.

In the multivariable logistic regression analysis, age, BMI, size of the fibroid, and fibroid multiplicity were adjusted for, and it was found that the laparoscopic approach was not an independent predictor of any complications (OR = 1.715, 95% CI: 0.283-10.378, p = 0.557; Table 8). None of the covariates were statistically significant, and this probably speaks to the low event rate.

**Table 8:** Multivariable logistic regression for complications OR > 1 indicates higher odds of the laparoscopic approach (or a per-unit increase). OR < 1 indicates lower odds.

Outcome / Predictor	Odds Ratio	95% CI Lower	95% CI Upper	P-value	Model Fit
Any Complication	-	-	-	-	Pseudo R² = 0.117, AIC = 63.9
Laparoscopic (vs. Open)	1.715	0.283	10.378	0.557	-
Age (years)	0.959	0.828	1.109	0.570	-
BMI (kg/m²)	0.863	0.710	1.049	0.139	-
Fibroid size (cm)	1.100	0.949	1.275	0.206	-
Multiple fibroids	0.774	0.127	4.704	0.781	-

Operation duration (Objective 4)

Laparoscopic myomectomy (median 213.0 minutes, IQR: 143.8-300.0) takes considerably more time than open myomectomy (median 121.0 minutes, IQR: 96.5-165.5; p < 0.001 (Table 2)). The effect size is large (rank-biserial r = −0.527), with a bootstrap 95% CI: 62-127 minutes (Table 4); median difference of 92 minutes. After multivariable adjustment, the laparoscopic approach was associated with a 90.5-minute increase in operative time (β = 90.490, 95% CI: 70.029-110.951; p < 0.001). Other independent predictors for time increase were larger fibroid size (β = 6.952 per cm, p < 0.001) and fibroid multiplicity (β = 45.256, p < 0.001; Table 5). However, for all examined subgroups, the difference in operative time was maintained. In patients with normal/underweight BMI, the median difference was 96 minutes (224 vs. 128.5 min; p < 0.001), whereas in overweight/obese patients, it was 92 minutes (213 vs. 121 min; p < 0.001). The difference was preserved in patients with a single fibroid (93.5 min difference; p < 0.001) and also in those with multiple fibroids (102.5 min difference; p < 0.001; Table 6).

Correlation analysis

In Figure 6, we detail the findings from Spearman's correlation analysis. Among other things, operative time is moderately positively correlated with estimated blood loss (ρ = 0.455, p < 0.05) and the size of the largest (most symptomatic) fibroid (ρ = 0.308, p < 0.05). Estimated blood loss also correlates positively with the size of the largest (most symptomatic) fibroid (ρ = 0.268, p < 0.05), length of stay (ρ = 0.286, p < 0.05), and drop in hemoglobin (ρ = 0.282, p < 0.05). In a blood loss multivariable regression analysis, BMI (β = 8.27, p = 0.013), fibroid size (β = 21.69, p < 0.001), and having multiple (≥2) fibroids (β = 125.34, p = 0.003) were independent predictors, while a surgical approach was not (mean difference (β) = -34.04, p = 0.470; Table 8). 

**Figure 6:**
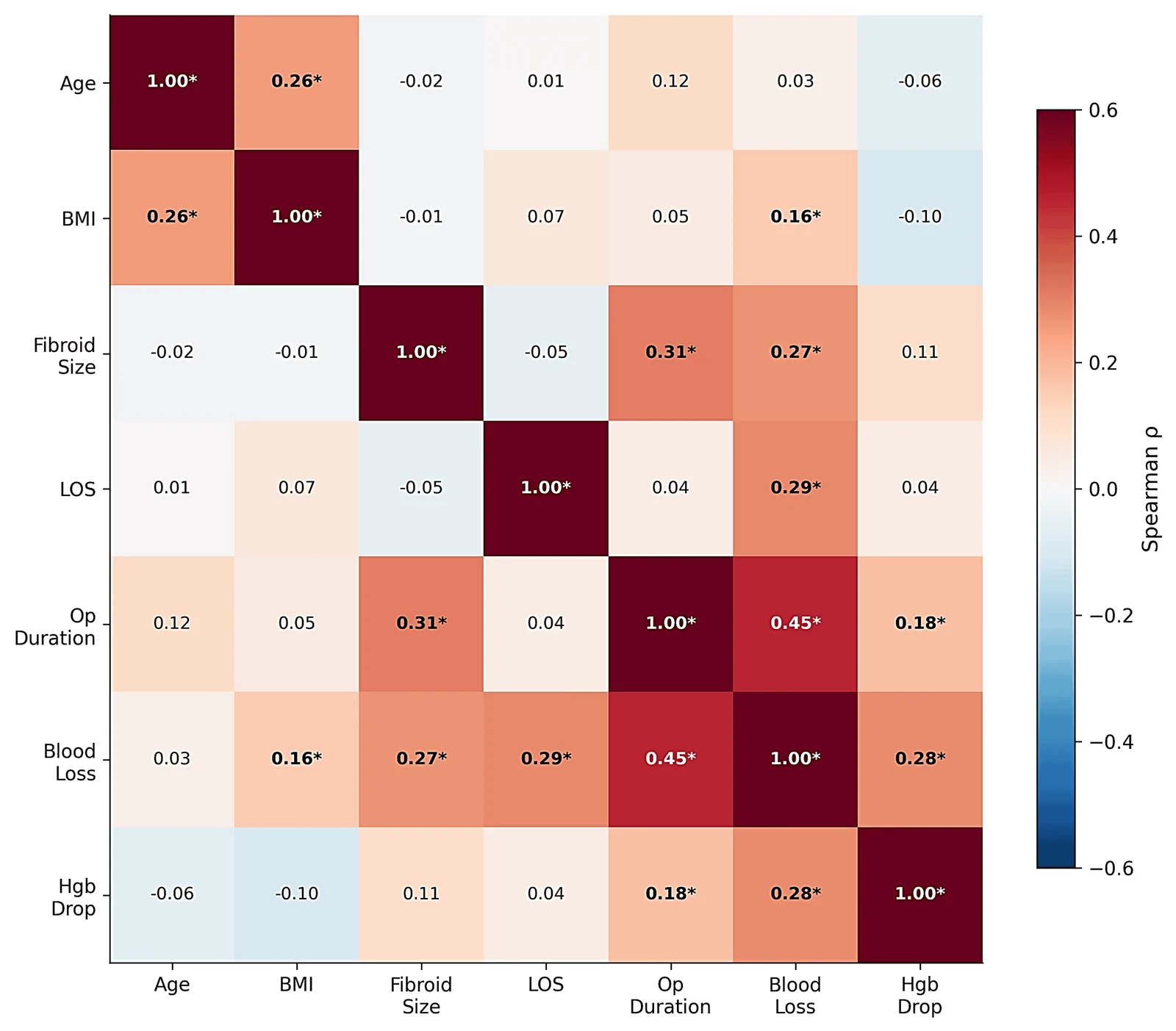
Spearman correlation matrix of key clinical variables Spearman correlation matrix of key clinical variables. Asterisks (*) denote statistically significant correlations (p < 0.05). The color scale ranges from −0.6 (blue) to +0.6 (red).

Temporal trends

Utilization of laparoscopic myomectomy was studied for the period 2021-2025, identifying trends in its adoption (Table 9, Figure 7). The share of laparoscopic techniques rose from 25.5% in 2021 to 37.9% in 2023, then declined to 22.6% in 2024 and 17.0% in 2025. The average adoption rate of the laparoscopic technique was 28.1% across the 2021-2025 period. The highest surgical volume was 77 procedures in 2022; the average for the years was about 60 procedures.

**Table 9:** Temporal trends in myomectomy approach (2021–2025) Data for 2025 may be incomplete due to the study cutoff (October 2025). Mean laparoscopic proportion across the study period: 28.1%.

Year	Total Procedures	Laparoscopic n (%)	Open n (%)	Laparoscopic Proportion (%)
2021	47	12 (25.5)	35 (74.5)	25.5
2022	77	29 (37.7)	48 (62.3)	37.7
2023	66	25 (37.9)	41 (62.1)	37.9
2024	62	14 (22.6)	48 (77.4)	22.6
2025	47	8 (17.0)	39 (83.0)	17.0

**Figure 7:**
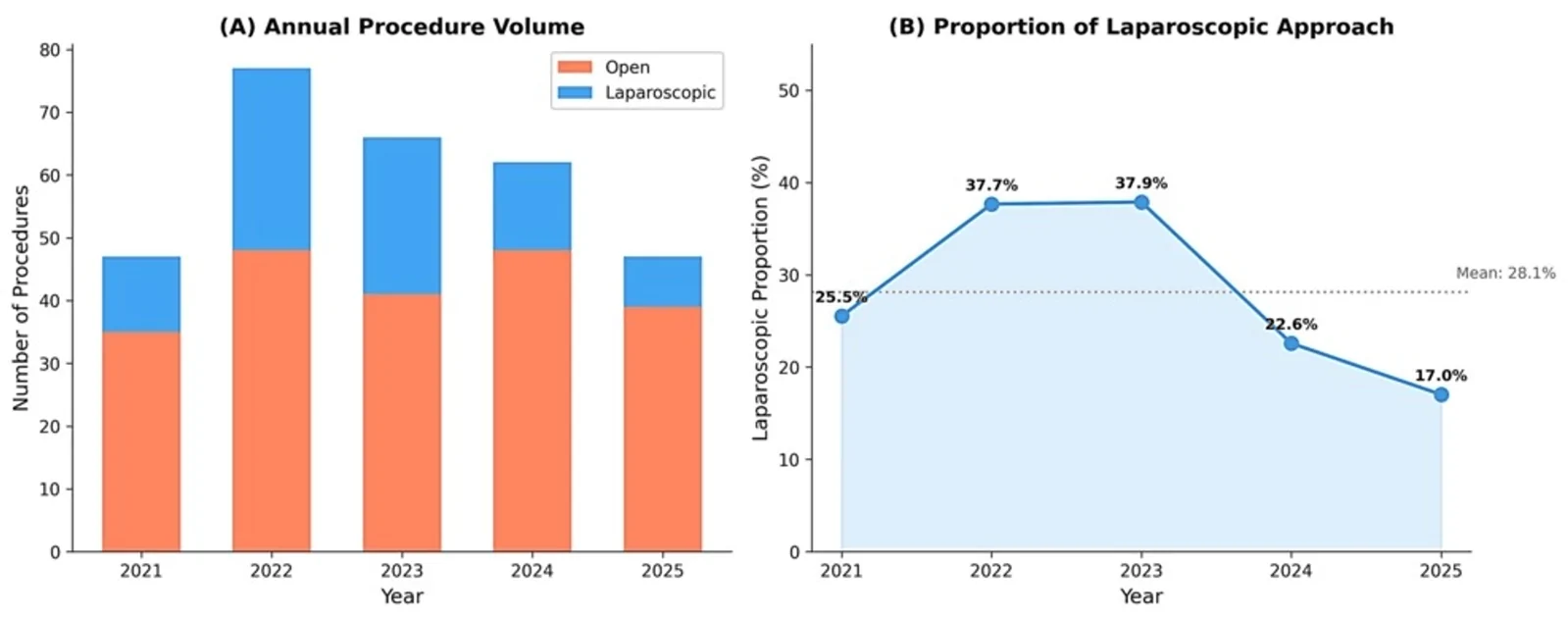
Temporal trends in myomectomy approach (2021–2025) A: annual procedure volume by type; B: proportion of laparoscopic procedures over time. The dashed line indicates the overall mean proportion (28.1%).

## Discussion

This retrospective study has provided valuable insights into the comparison between the surgical outcomes of laparoscopic myomectomy and open myomectomy in women with fibroids. Laparoscopic myomectomy was associated with a statistically significant shorter hospital stay than open myomectomy; however, it required significantly longer operative time, reflecting the technical demands of a minimally invasive approach. For outcomes such as estimated blood loss, hemoglobin reduction, and postoperative complications, this study found no significant differences between the two approaches. Similarly, Giannini et al.'s findings describe that when comparing laparoscopic myomectomy to open myomectomy, laparoscopic myomectomy has a shorter length of stay and has no statistically significant difference when it comes to postoperative complications [10]. In contrast, Tanos et al. report that laparoscopic myomectomy is associated with less blood loss, hemoglobin blood loss, and a lower incidence of postoperative fever [11]. Also, Ibrahim et al.'s systematic review reported that there is a significant reduction in blood loss intraoperatively with the laparoscopic approach in comparison to the open approach [12]. Of particular note, in relation to the characteristics of the fibroids, this study found that size and number were much more important than the surgical approach in determining blood loss and surgical complexity. Han et al.'s finding supports that fibroid characteristics had an impact on postoperative blood loss [13]. However, Casarin et al.'s findings demonstrated that fibroid characteristics had no effect on the risk of complications [14].

This study contains some limitations. First, the retrospective design introduces the possibility of information bias. Second, since the study was limited to a single specialized tertiary women's hospital, the findings may limit the generalizability of the results to other healthcare settings with less expertise and fewer resources. Third, there may have been selection bias because the surgical technique was chosen primarily on surgeon preference and clinical judgment rather than randomization. For instance, open surgery was more commonly used to treat individuals with multiple fibroids. Additionally, the statistical balance of comparisons may have been impacted by the different sample sizes between the open and laparoscopic groups. Finally, even though the analysis adjusted for several significant confounders, such as age, body mass index, fibroid size, and fibroid multiplicity, other potentially relevant factors such as surgeon experience and intraoperative techniques were not available in the dataset and therefore could not be accounted for in the analysis.

The study’s setting took place at a tertiary specialized women’s hospital, which provided a homogeneous patient population and reduced possible confounding variables. Additionally, the relatively large sample size increases the significance of our findings and their application to similar clinical settings. Furthermore, the use of a random sampling technique minimized selection bias by ensuring that all eligible participants had an equal probability of inclusion.

Overall, the results support laparoscopic myomectomy as a surgical approach that provides an advantage when it comes to reduced hospitalization. Regarding morbidity and blood loss, there were no significant differences between open and laparoscopic myomectomy. The setting of the study, sample size, and random sampling technique affirm the significance of the study's main findings and support laparoscopic myomectomy as a safe surgical option.

## Conclusions

This study reveals that laparoscopic myomectomy was associated with a shorter hospital stay, affirming its role as a minimally invasive approach that facilitates postoperative recovery in appropriately selected women with uterine fibroids. Of note, open myomectomy and laparoscopic myomectomy yielded similar results in terms of intraoperative blood loss, hemoglobin loss, and postoperative complications. In conclusion, laparoscopic myomectomy was associated with shorter hospitalization and longer operative time, without statistically detectable differences in the measured perioperative complications.
